# Increased risks of type II diabetes among older Asian, Native Hawaiian, and Pacific Islander lung cancer survivors: a SEER-Medicare analysis

**DOI:** 10.1007/s11764-025-01786-5

**Published:** 2025-03-29

**Authors:** Yihang Freya Zhang, Chun-Pin Esther Chang, Thomas K. Varghese, Randa Tao, Mia Hashibe

**Affiliations:** 1 Department of Epidemiology, UCLA Fielding School of Public Health, Los Angeles, CA USA; 2 Division of Public Health, Department of Family and Preventive Medicine, University of Utah School of Medicine, Salt Lake City, UT USA; 3 Huntsman Cancer Institute, University of Utah, 2000 Circle of Hope, Salt Lake City, UT 84108 USA; 4 Division of Cardiothoracic Surgery, Department of Surgery, University of Utah, Salt Lake City, UT USA; 5 Department of Radiation Oncology, University of Utah School of Medicine, Salt Lake City, UT USA; 6 Mayo Clinic, Phoenix, AZ USA

**Keywords:** Asian, Native Hawaiian, And Pacific Islander, Lung cancer survivors, Diabetes; Health disparities

## Abstract

**Purpose:**

Asian, Native Hawaiian, and Pacific Islanders (ANHPI) have a higher prevalence of type II diabetes compared to the non-Hispanic White (NHW) population. However, the incidence of type II diabetes among ANHPI lung cancer survivors is unknown. The aim of this study is to investigate the risk of newly diagnosed type II diabetes among older ANHPI lung cancer survivors compared to older NHW lung cancer survivors.

**Methods:**

We identified 3920 ANHPI and 11,760 NHW lung cancer survivors diagnosed from 2000 to 2017 from the Surveillance, Epidemiology, and End Results (SEER)-Medicare database. Cox proportional hazard models were used to estimate the hazard ratios (HRs) and 95% confidence intervals (CIs) for the risk of type II diabetes, adjusting for potential confounding factors.

**Results:**

Older ANHPI lung cancer survivors had an increased risk of incident type II diabetes compared to older NHW lung cancer survivors > 1 year after cancer diagnosis. For specific ANHPI subgroups, increased risks of type II diabetes were observed among Asian Indian and Pakistani (HR, 5.14; 95% CI 2.10, 12.60) compared to NHW patients, followed by Native Hawaiian and Pacific Islander (NHPI) (HR, 4.38; 95% CI 1.22, 15.74), Vietnamese (HR, 2.32; 95% CI 1.29, 4.18), Korean (HR, 2.26; 95% CI 1.13, 4.54), Filipino (HR, 1.78; 95% CI 1.00, 3.16), and Chinese (HR, 1.75; 95% CI 1.10, 2.79) lung cancer survivors.

**Conclusion:**

Older ANHPI lung cancer survivors had a higher risk of type II diabetes compared to older NHW lung cancer survivors. Further research is needed to examine the underlying causes of health disparities in diabetes risk among ANHPI lung cancer survivors.

**Implications for Cancer Survivors:**

These findings suggest that increasing surveillance on type II diabetes and monitoring body weight for older ANHPI lung cancer patients may be beneficial.

**Supplementary Information:**

The online version contains supplementary material available at https://doi.org/10.1007/s11764-025-01786-5.

## Introduction

Lung cancer is the second most common cancer and the leading cause of death in the United States (US) [1]. In 2024, an estimated 238,480 people in the US were diagnosed with lung cancer, and 127,070 died from the disease [1]. The 5-year survival rate of lung cancer was 25.4% for individuals diagnosed in 2013 to 2019 [2]. As of 2022, there were approximately 654,620 lung cancer survivors in the US, with a median diagnosis age of 71 years old [3]. Among Asian, Native Hawaiians, and Pacific Islander (ANHPI) population, lung cancer ranks as the second most common cancer and is the leading cause of cancer death [4]. ANHPI individuals were more likely to be diagnosed with lung cancer at a distant cancer stage compared to Non-Hispanic White (NHW) individuals [4]. The 5-year survival rates for ANHPIs with lung cancer were 22.7% for males and 30.6% for females diagnosed in 2001–2016 [5]. Among ANHPI lung cancer patients, the 5-year survival rates were 37% for Thai, 36% for Chinese, 35% for Pakistani, 29% for Asian Indian, 28% for Korean, 27% for Filipino, 27% for Vietnamese, 23% for Native Hawaiian, and 21% for Japanese lung cancer patients reported in 2023 [6].

Diabetes is one of the health concerns for cancer survivors [7]. A meta-analysis study reported that the risk of diabetes was 1.39 times higher among cancer survivors than individuals without cancer in the general population (95% CI 1.29, 1.50) [8]. The prevalence of diabetes among lung cancer patients was approximately 18.3% [9]. A cohort study in Korea including 1065 lung cancer patients reported that lung cancer patients had a 1.74-fold risk of type II diabetes (95% CI 1.34, 2.24) compared to the cancer-free population [10]. Another study including 2489 women with lung cancer reported that lung cancer patients with diabetes had a 27% higher risk of death compared to patients without diabetes (95% CI 1.07, 1.50) [11]. The mechanism of diabetes risks among lung cancer patients is not yet fully understood. Previous studies suggest that chemotherapy, such as cisplatin, may induce insulin resistance and hyperglycemia, which are associated with an increased risk of diabetes in cancer patients [12]. Lung cancer patients treated with immunotherapies, such as PD/PD-L1 and CTLA-4 inhibitors, and targeted therapies, like PI3K/mTOR inhibitors, may be at increased risk of developing type II diabetes due to reduced insulin production and insulin resistance. Additionally, corticosteroids, commonly used in cancer treatment, could impair insulin sensitivity, further contributing to the risk of type II diabetes [13]. While previous studies have shown that the ANHPI population had a higher prevalence of type II diabetes compared to NHW population [14], the risk of diabetes among ANHPI lung cancer survivors remains understudied.

To our knowledge, no study has been conducted to investigate the risk of incident type II diabetes among ANHPI lung cancer survivors compared to NHW lung cancer survivors. This study aimed to examine the risk of incident type II diabetes after cancer diagnosis among older ANHPI lung cancer survivors in comparison with the older NHW lung cancer survivors and to assess risk factors of type II diabetes among older lung cancer survivors.

## Methods

The Surveillance, Epidemiology, and End Results (SEER)-Medicare database links two large population-based sources of data that provide detailed information about Medicare beneficiaries with cancer [15]. The SEER data includes demographic characteristics and cancer-related clinical information, such as age at cancer diagnosis, sex, race, ethnicity, cancer site, cancer stage, and the first-course cancer treatment [16]. The Medicare data provides the information on Medicare enrollment and claims from outpatient, inpatient, and physician services [17]. This study was deemed exempt by the Institutional Review Board at the University of Utah.

We included patients who were (1) ANHPI or NHW, (2) aged ≥ 66 years old at cancer diagnosis, (3) diagnosed between 2000 and 2017, and (4) diagnosed with the first primary invasive lung cancer (SEER Site code: 22030). Patients were excluded if they met any of the following criteria: (1) not being continuously enrolled in Medicare Part A or Part B or being enrolled in Health Maintenance Organization (HMO) from 1 year prior to cancer diagnosis to the end of SEER follow-up period (*N* = 120,883), (2) having less than 1 year of follow-up (*N* = 124,804), (3) being identified by autopsy or death certificate (*N* = 373), and (4) having unusual histology types (*N* = 601) (Supplemental Fig. 1). To ensure a full year of enrollment before cancer diagnosis for baseline comorbidity calculation, we included patients diagnosed at 66 or older. Each ANHPI lung cancer survivor was matched to three NHW lung cancer survivors by sex, year of cancer diagnosis, and age at cancer diagnosis. The final analysis included 3920 ANHPI and 11,760 NHW lung cancer survivors.

We used the race/ethnicity information from the SEER to categorize ANHPI subgroup: East Asian included Chinese, Japanese, or Korean; Southeast Asian included Filipino, Vietnamese, Laotian, Hmong, Kampuchean, or Thai; South Asian included Asian Indian and/or Pakistani; Native Hawaiian and Pacific Islander (NHPI) included Micronesian, Chamorran, Guamanian, Polynesian, Tahitian, Samoan, Tongan, Melanesian, Fiji Islander, New Guinean, or Pacific Islander. International Classification of Diseases (ICD)−9 and 10 diagnosis codes were used to identify type II diabetes diagnoses. Patients were considered to have a type II diabetes diagnosis if the corresponding ICD code appeared in one claim of inpatient claims, or at least two claims of outpatient or physician claims within 30–60 days to avoid overestimation. Patients diagnosed with diabetes at any time up to 1 year after their cancer diagnosis were excluded from the analysis as prevalent when we estimated the risk of incident diabetes. The modified Charlson Comorbidity Index (CCI) was calculated to assess baseline comorbidities within a year prior to cancer diagnosis, excluding cancers and diabetes [18] to avoid overadjustment. The Rural–Urban Continuum Codes (RUCC) available in the SEER data were used to classify rural and urban residence status [19]. To ensure that rural–urban classification reflects the time of diagnosis, we applied the RUCC corresponding to the cancer diagnosis year: RUCC 2003 for diagnoses from 2000 to 2008 and RUCC 2013 for diagnoses in 2009 and later. Census tract education and income at cancer diagnosis were available. Obesity at cancer diagnosis was obtained from Chronic Conditions Warehouse [20] using ICD-9 and 10 code to capture obesity diagnosis in both ANHPI and NHW lung cancer survivors. The follow-up period was calculated from lung cancer diagnosis to the diagnosis of diabetes, death, or the last date of follow-up, whichever occurred first. First-course cancer treatment, including surgery, radiotherapy, and chemotherapy, was represented by the proportion of patients undergoing each treatment. Patients may have received more than one treatment. The SEER registries were categorized registries into four geographic areas: West (San Francisco-Oakland, Hawaii, New Mexico, Seattle-Puget Sound, Utah, San Jose-Monterey, Los Angeles, Greater California, Idaho), South (Metropolitan Atlanta, Rural Georgia, Kentucky, Louisiana, Greater Georgia), Northeast (Connecticut, New Jersey, New York, Massachusetts), and Midwest (Metropolitan Detroit, Iowa).

### Statistical analysis

Chi-square tests were used to examine the distribution of demographic and clinical characteristics between ANHPI and NHW lung cancer survivors. Cox proportional hazard models were used to estimate the hazard ratios (HRs) and 95% confidence intervals (CIs) for the risk of type II diabetes among ANHPI lung cancer survivors compared to NHW lung cancer survivors [21]. We also used Chinese lung cancer patients as the reference group to compare the HRs among the ANHPI subgroups and specific races and ethnicities. Proportional hazard assumptions were tested for all Cox proportional hazard models [22]. If the proportional hazard assumption was violated, the flexible parametric survival model with cubic spline functions was used to calculate HRs and 95% CIs [23]. Cox proportional hazard models were also used to examine risk factors for type II diabetes among lung cancer survivors. Potential risk factors included ANHPI subgroup, age of cancer diagnosis, SEER registry region, census tract education, census tract income, rural residence, baseline CCI, cancer stage, and cancer treatments. To account for potential confounding, we adjusted for baseline CCI, SEER registry region, rural residence, census tract education, and census tract income, based on three properties of confounding factors and causal diagram [24]. These factors are potential risk factors for T2DM and are associated with race and ethnicity [25–27]. For example, baseline CCI may increase the risk of T2DM. Moreover, the distribution of baseline CCI differs between ANHPI and NHW lung cancer survivors. Education and income may act as mediators; however, we chose to adjust for these factors to estimate the association independent of socioeconomic status. The statistical analyses were conducted using SAS version 9.4 (SAS Institute, Inc., Cary, NC) and Stata version 14 (StataCorp LLC, College Station, TX). All *p*-values were calculated as two-sided, and any *p*-values below the threshold of 0.05 were deemed to be statistically significant.

## Results

There were 3665 Asian, 255 NHPI, and 11,760 NHW lung cancer survivors in the final analysis (Table 1). Compared to NHW lung cancer survivors, Asian lung cancer survivors had a higher percentage of higher census tract education (16.1% vs. 12.8%, *p* < 0.001), higher census tract income (20.5% vs. 14.8%, *p* < 0.001), and CCI score of 0 at cancer diagnosis (49.6% vs. 37.0%, *p* < 0.001). A lower percentage of Asian lung cancer survivors resided in rural areas (2.9% vs. 18.8%, *p* < 0.001) or had obesity at cancer diagnosis (3.5% vs. 9.9%, *p* < 0.001) compared to NHW lung cancer survivors. Compared to NHW lung cancer survivors, NHPI lung cancer survivors had lower census tract income (9.4% with income of $90,000 +), were more likely to live in rural areas (23.5%), and had a higher percentage of obesity (11.4%). NHPI lung cancer survivors had a younger mean age at the time of cancer diagnosis (74 years) in comparison to Asian lung cancer survivors (76 years).

**Table 1:** Demographic characteristics of lung cancer survivors diagnosed from 2000 to 2017 in the SEER-Medicare data, stratified by race and ethnicity

	Asian (*n* = 3665)		NHPI (*n* = 255)		NHW (*n* = 11,760)		*p*-value^a^
	*n*	%	*n*	%	*n*	%	
Age of cancer diagnosis							0.001
66–70	800	21.8	79	31.0	2637	22.4	
71–75	1026	28.0	77	30.2	3309	28.1	
76–80	944	25.8	65	25.5	3027	25.8	
81–100 +	895	24.4	34	13.3	2787	23.7	
Age of cancer diagnosis, mean (SD)	76 (6.2)		74 (5.6)		76 (6.2)		
Sex							0.894
Male	1841	50.2	132	51.8	5919	50.3	
Female	1824	49.8	123	48.2	5841	49.7	
Census tract education^b^							< 0.001
≤ 40% had some college education	506	13.8	37	14.5	2068	17.6	
40 to ≤ 60% had some college education	1178	32.1	122	47.8	4250	36.1	
60 to ≤ 80% had some college education	--^c^	--^c^	--^c^	--^c^	--^c^	--^c^	
> 80% had some college education	589	16.1	--^c^	--^c^	1500	12.8	
Census tract income^b^							< 0.001
< $50,000	1169	31.9	95	37.2	5040	42.9	
$50,000 to < $70,000	1001	27.3	83	32.6	3191	27.1	
$70,000 to < $90,000	--^c^	--^c^	53	20.8	--^c^	--^c^	
$90,000 +	750	20.5	24	9.4	1736	14.8	
RUCC							< 0.001
Urban	3560	97.1	195	76.5	9545	81.2	
Rural	105	2.9	60	23.5	2215	18.8	
Follow-up years							0.320
> 1 to 5	2814	76.8	195	76.5	8890	75.6	
> 5 to 10	637	17.4	47	18.4	2223	18.9	
> 10	214	5.8	13	5.1	647	5.5	
Modified Charlson Comorbidity Index^d^							< 0.001
0	1816	49.6	118	46.3	4348	37.0	
1	1063	29.0	80	31.4	3881	33.0	
2 +	786	21.4	57	22.3	3531	30.0	
Obesity at cancer diagnosis							< 0.001
No	3538	96.5	226	88.6	10,601	90.1	
Yes	127	3.5	29	11.4	1159	9.9	

*Abbreviations*: *NHPI*, Native Hawaiian and Pacific Islander; *NHW*, Non-Hispanic White; *RUCC*, Rural–Urban Commuting Area Codes

^a^*p* for chi-square

^b^Unknown: < 11

^c^Counts (< 11) and % were suppressed per US Department of Health and Human Services CMS cell size suppression policy. Additional cells are suppressed so suppressed numbers cannot be calculated

^d^Excluded diabetes and cancer

Asian (41.1%) and NHPI (33.7%) lung cancer patients were more likely to be diagnosed at distant stage compared to NHW lung cancer patients (29.1%, Table 2). In terms of cancer treatment, Asian (35.5%) and NHPI (35.7%) lung cancer survivors were less likely to receive surgery in comparison to NHW lung cancer survivors (40.1%, *p* < 0.001). In contrast, Asian (44.8%) and NHPI (45.1%) lung cancer patients were both more likely to receive chemotherapy compared to NHW lung cancer patients (38.8%, *p* < 0.001). Regarding the cancer treatment among the ANHPI subgroup, South Asian patients (42.0%) were more likely to receive surgery while Southeast Asian patients (30.5%) were less likely to receive surgery; East Asian (33.5%) and Southeast Asian (33.6%) patients were less likely to receive radiotherapy; East Asian (45.9%), Southeast Asian (45.4%), and NHPI (45.1%) patients were more likely to receive chemotherapy (Supplemental Fig. 2). In terms of histology type, a higher percentage (62.0%) of Asian lung cancer survivors were diagnosed with adenocarcinoma in comparison to NHPI lung cancer survivors (46.3%) and NHW lung cancer survivors (44.5%) (Table 2).

**Table 2:** Clinical characteristics of lung cancer survivors diagnosed from 2000 to 2017 in the SEER-Medicare data, stratified by race and ethnicity

	Asian (*n* = 3665)		NHPI (*n* = 255)		NHW (*n* = 11,760)	
	*n*	%	*n*	%	*n*	%
Cancer stage^a^						
Localized	1105	30.1	74	29.0	4466	38
Regional	1033	28.2	95	37.3	3865	32.9
Distant	1527	41.7	86	33.7	3429	29.1
AJCC^a^						
I	1072	29.2	87	34.1	4314	36.7
II	194	5.3	78	30.6	626	5.3
III	864	23.6	45	17.6	2622	22.3
IV	908	24.8	--^b^	--^b^	2128	18.1
Unknown	627	17.1	--^b^	--^b^	2070	17.6
Received surgery^a,c^	1299	35.5	91	35.7	4715	40.1
Received radiotherapy^a,c^	1221	33.3	111	43.5	4597	39.1
Received chemotherapy^a,c^	1642	44.8	115	45.1	4565	38.8
Histology type^a^						
Adenocarcinoma	2271	62.0	118	46.3	5237	44.5
Squamous cell carcinoma	572	15.6	68	26.7	3087	26.3
Small cell carcinoma	181	4.9	20	7.8	975	8.3
Non-small cell carcinoma	289	7.9	22	8.6	921	7.8
Large cell carcinoma	71	2.0	--^b^	--^b^	258	2.2
Unspecified malignant neoplasms	118	3.2	--^b^	--^b^	385	3.3
Malignant neoplasms NOS	70	1.9	--^b^	--^b^	335	2.9
Other specified carcinoma	93	2.5	--^b^	--^b^	562	4.8

*Abbreviations*: *NHPI*, Native Hawaiian and Pacific Islander; *NHW*, Non-Hispanic White; *AJCC*, American Joint Committee on Cancer; *NOS*, not otherwise specified

^a^*p*-value for chi-square < 0.001

^b^Counts (< 11) and % were suppressed per US Department of Health and Human Services CMS cell size suppression policy. Additional cells are suppressed so suppressed numbers cannot be calculated

^c^Patients may have received more than one treatment. We presented the percentage of receiving each treatment. Thus, they are not mutually exclusive

The prevalence of type II diabetes before cancer diagnosis and up to 1 year after cancer diagnosis was 43.4% among Filipino, 42.7% among Korean, 40.4% among Vietnamese, 39.9% among Asian Indian and Pakistani, and 25.9% among NHW lung cancer survivors (Supplemental Table 1). When grouped, 42.1% of Southeast Asians, 39.9% of South Asians, 36.1% of NHPIs, and 32.3% of East Asians had a diagnosis of prevalent type II diabetes before cancer diagnosis and up to 1 year after cancer diagnosis (Supplemental Table 2).

Asian Indian and Pakistani lung cancer survivors had a higher risk of type II diabetes (HR, 5.14; 95% CI 2.10, 12.60) compared to NHW patients > 1 year after lung cancer diagnosis, followed by NHPI (HR, 4.38; 95% CI 1.22, 15.74), Vietnamese (HR, 2.32; 95% CI 1.29, 4.18), Korean (HR, 2.26; 95% CI 1.13, 4.54), Filipino (HR, 1.78; 95% CI 1.00, 3.16), and Chinese (HR, 1.75; 95% CI 1.10, 2.79) lung cancer survivors (Table 3). Compared to Chinese lung cancer survivors, a higher risk of type II diabetes was observed for Asian Indian and Pakistani lung cancer survivors (HR, 2.93; 95% CI 1.08, 7.99). Within the Asian lung cancer survivors, an increased risk of type II diabetes in South Asian (HR, 4.92; 95% CI 2.03, 11.94), Southeast Asian (HR, 1.84; 95% CI 1.20, 2.82), and East Asian (HR, 1.74; 95% CI 1.20, 2.53) was observed compared to NHW (Supplemental Table 3). When stratified by sex, an increased risk of type II diabetes was observed among male and female ANHPI lung cancer survivors compared to NHW lung cancer survivors (HR_male_, 2.22; 95% CI 1.39, 3.56; HR_female_, 1.91; 95% CI 1.30, 2.80; Supplemental Table 4). Among Asian lung cancer survivors, Asian Indian and Pakistani individuals had a higher risk of type II diabetes for both males and females (Supplemental Table 4). Chinese and Japanese male lung cancer survivors had a higher risk of type II diabetes compared to NHW male lung cancer survivors, while Filipino, Vietnamese, and Korean female lung cancer survivors had a higher risk of type II diabetes compared to NHW female lung cancer survivors (Supplemental Table 4).

**Table 3:** The risks of type II diabetes among Asian lung cancer survivors > 1 year after cancer diagnosis

	Diabetes		No diabetes		HR (95% CI)^a^	HR (95% CI)^a^
	***N***	**%**	***N***	**%**		
Race/ethnicity						
NHW	448	8.0	5176	92.0	Reference	**0.57 (0.36, 0.91)**
Chinese	76	10.2	667	89.8	**1.75 (1.10, 2.79)**	Reference
Filipino	43	12.0	316	88.0	**1.78 (1.00, 3.16)**	1.02 (0.51, 2.01)
Japanese	31	7.0	411	93.0	1.31 (0.70, 2.47)	0.75 (0.36, 1.54)
Vietnamese	44	17.3	210	82.7	**2.32 (1.29, 4.18)**	1.32 (0.67, 2.60)
Other Asian	25	11.0	203	89.0	1.52 (0.80, 2.90)	0.87 (0.42, 1.80)
Korean	29	14.7	169	85.3	**2.26 (1.13, 4.54)**	1.29 (0.60, 2.78)
Asian Indian and Pakistani	24	29.3	58	70.7	**5.14 (2.10, 12.60)**	**2.93 (1.08, 7.99)**
NHPI	11	6.9	148	93.1	**4.38 (1.22, 15.74)**	2.50 (0.66, 9.44)

*Abbreviations*: *NHW*, Non-Hispanic White; *NHPI*, Native Hawaiian and Pacific Islander; *HR*, hazard ratio; *CI*, confidence interval

^a^Models adjusted for baseline Charlson Comorbidity Index, SEER registry region, rural residence, census tract education, and census tract income

Bold text indicates statistical significance

**Table 4:** The risks of type II diabetes among lung cancer survivors > 1 year after cancer diagnosis, stratified by race and ethnicity

	ANHPI (*n* = 2512^a^)				HR (95% CI)	NHW (*n* = 8710^a^)				HR (95% CI)
	Diabetes (*n* = 285)		No diabetes (*n* = 2227)			Diabetes (*n* = 656)		No diabetes (*n* = 8054)		
	*n*	%	*n*	%		***n***	%	*n*	%	
Census tract education^b^										
≤ 40% had some college education	46	13.2	302	86.8	Reference	131	8.9	1338	91.1	Reference
40 to ≤ 60% had some college education	98	12.1	714	87.9	1.00 (0.68, 1.46)	261	8.5	2794	91.5	0.94 (0.75, 1.91)
60 to ≤ 80% had some college education	114	12.0	835	88.0	0.98 (0.64, 1.50)	203	6.8	2782	93.2	**0.69 (0.52, 0.93)**
> 80% had some college education	26	6.5	375	93.5	**0.50 (0.27, 0.93)**	59	4.9	1137	95.1	**0.48 (0.31, 0.73)**
Modified CCI^c^										
0	152	11.2	1200	88.8	Reference	254	7.2	3266	92.8	Reference
1	90	12.2	647	87.8	0.99 (0.76, 1.30)	221	7.5	2729	92.5	1.10 (0.91, 1.31)
2 +	43	10.2	380	89.8	0.94 (0.66, 1.34)	181	8.1	2059	91.9	**1.33 (1.09, 1.61)**
Obesity at cancer diagnosis^d^										
No	--^f^	--^f^	--^f^	--^f^	Reference	604	7.4	7558	92.6	Reference
Yes	--^f^	--^f^	--^f^	--^f^	**2.11 (1.02, 4.34)**	52	9.5	496	90.5	**1.41 (1.06, 1.88)**
Cancer stage^d^										
Localized	110	15.0	625	85.0	Reference	315	9.6	2978	90.4	Reference
Regional	89	12.5	621	87.5	1.05 (0.79, 1.39)	222	7.8	2610	92.2	1.04 (0.88, 1.24)
Distant	86	8.1	981	91.9	1.13 (0.84, 1.53)	119	4.6	2466	95.4	1.17 (0.94, 1.46)
Received surgery^e^										
No	141	8.8	1460	91.2	Reference	280	5.5	4844	94.5	Reference
Yes	144	15.9	762	84.1	0.88 (0.65, 1.19)	373	10.5	3165	89.5	**0.83 (0.69, 0.99)**
Received radiotherapy^e^										
None/unknown	205	12.2	1480	87.8	Reference	440	8.2	4892	91.8	Reference
Yes	80	9.7	747	90.3	1.05 (0.80, 1.38)	216	6.4	3162	93.6	1.11 (0.93, 1.32)
Received chemotherapy^e^										
None/unknown	169	12.2	1218	87.8	Reference	443	8.3	4868	91.7	Reference
Yes	116	10.3	1009	89.7	1.25 (0.94, 1.67)	213	6.3	3186	93.7	1.08 (0.89, 1.31)

*Abbreviations*: *ANHPI*, Asian, Native Hawaiian and Pacific Islander; *NWH*, Non-Hispanic White; *HR*, hazard ratio; *CI*, confidence interval; *CCI*, Charlson Comorbidity Index

^a^Removed patients diagnosed with diabetes before the study time period (> 1 year after cancer diagnosis)

^b^Model adjusted for sex, race, SEER registry region, baseline CCI, age of cancer diagnosis, census tract income, and rural residence

^c^Model adjusted for sex, race, SEER registry region, age of cancer diagnosis, census tract income, census tract education, and rural residence

^d^Model adjusted for sex, race, SEER registry region, age of cancer diagnosis, census tract income, census tract education, rural residence, and baseline CCI

^e^Models adjusted for sex, race, SEER registry region, age of cancer diagnosis, census tract income, census tract education, rural residence, baseline CCI, and cancer stage

^f^Counts (< 11) and % were suppressed per US Department of Health and Human Services CMS cell size suppression policy. Additional cells are suppressed so suppressed numbers cannot be calculated

Bold text indicates statistical significance

Among ANHPI lung cancer survivors, individuals who were obese at cancer diagnosis had a 2.11-fold higher risk of developing type II diabetes compared to those who were not obese. Similarly, a 1.41-fold higher risk was observed among NHW lung cancer survivors who were obese at cancer diagnosis (Table 4). Lung cancer survivors with the highest education level had a lower risk of type II diabetes for both ANHPI (HR, 0.50; 95% CI 0.27, 0.93) and NHW (HR, 0.48; 95% CI 0.31, 0.73). Among NHW lung cancer survivors, a modified CCI score of 2 + was associated with a higher risk of type II diabetes (HR, 1.33; 95% CI 1.09, 1.61), whereas no such association was found in ANHPI lung cancer survivors (HR, 0.94; 95% CI 0.66, 1.34). Surgical treatment was associated with a lower risk of type II diabetes in NHW lung cancer survivors (HR, 0.83; 95% CI 0.69, 0.99). We did not observe an association between type II diabetes risk and census tract income, cancer stage, and cancer treatment among ANHPI lung cancer survivors. An association between the AJCC stage and the risk of type II diabetes was not observed (data not shown). Supplemental Table 5 presents the percentage of type II diabetes by age, sex, race, and ethnicity, regardless of the prevalent type II diabetes.

## Discussion

In our analysis using SEER-Medicare data, we observed a higher prevalence and incidence of type II diabetes among older ANHPI lung cancer survivors compared to older NHW lung cancer survivors. This was despite a lower incidence of obesity in ANHPI compared to NHW patients at cancer diagnosis. Both Asian and NHPI lung cancer survivors had higher risks of type II diabetes > 1 year after cancer diagnosis compared to NHW lung cancer survivors. For Asian lung cancer survivors, South Asian lung cancer survivors had the highest incidence of type II diabetes. In a more detailed breakdown, Asian Indian and Pakistani lung cancer survivors had the highest incidence, followed by those of Vietnamese, Korean, Vietnamese, Filipino, and Chinese descent. The variation within the ANHPI subgroup highlights the importance of disaggregating data. It is crucial to understand the type II diabetes risk within ANHPI subgroups after lung cancer diagnosis in order to tailor healthcare interventions and resources effectively. ANHPI lung cancer survivors had a higher percentage of having distant metastatic disease at the time of diagnosis compared to NHW lung cancer survivors. This observation might reflect potential delays in cancer screening [28], limited access to healthcare, or systemic inequalities in the diagnostic process among ANHPI lung cancer survivors that can indirectly influence higher diabetes risk. When stratified by sex, no consistent pattern was observed across ANHPI lung cancer survivor subgroups for type II diabetes risks. However, the limited sample size in each subgroup reduces the reliability of the findings, emphasizing the need for further research.

South Asian and NHPI lung cancer survivors had a more than fourfold higher risk of type II diabetes compared to NHW lung cancer survivors. This association may be attributed to a complex interplay of genetics, lifestyle, healthcare access, and cultural factors. South Asians, including those of Asian Indian and Pakistani descent, may have a genetic predisposition to type II diabetes, characterized by reduced beta-cell function and impaired insulin action [29]. In addition, traditional diets in South Asian cultures often include high-carbohydrate and sugar-rich foods, which can contribute to obesity and metabolic syndrome, both of which are risk factors for type II diabetes [30]. The cultural preference for certain types of food that are high in trans fats and sugars may lead to a higher prevalence of these conditions [31]. For the NHPI population, a combination of barriers including inadequate healthcare access, economic instability, and significant shifts in nutrition and lifestyle due to colonization and environmental changes might contribute to the high rates of diabetes within this community [32]. Variations in type II diabetes risks among different ANHPI subgroups suggest that targeted interventions, culturally competent care, and further research to uncover the specific mechanisms underlying the increased diabetes risk within this diverse population are needed.

ANHPI lung cancer survivors with obesity at cancer diagnosis had a twofold higher risk of developing type II diabetes compared to those without obesity. Obesity is a risk factor of type II diabetes in the general population [33], and our finding highlights the importance of weight management in preventing type II diabetes among ANHPI lung cancer survivors. In our study, higher education was associated with a decreased risk of type II diabetes among lung cancer survivors. One reason could be that higher education is associated with better health literacy, diabetes knowledge, and self-care activities, as they may lead to reductions in fasting blood glucose, body mass index, and waist circumference [34]. Among NHW lung cancer survivors, a modified CCI score of 2 + was linked to higher type II diabetes risk, likely due to better healthcare access, enabling more effective management of comorbidities. In contrast, baseline ANHPI lung cancer survivors may face healthcare barriers that limit comorbidity management, explaining the lack of association [35]. Surgical treatment was associated with a decreased type II diabetes risk in NHW lung cancer survivors. However, this inverse association was not observed in ANHPI lung cancer survivors, potentially due to a limited sample size. Although ANHPI lung cancer survivors were more likely to receive chemotherapy compared to NHW lung cancer survivors, we did not observe an association between chemotherapy and type II diabetes in either group. Previous studies suggest that chemotherapy agents such as 5-fluorouracil and cisplatin can induce insulin resistance and hyperglycemia, leading to an increased risk of diabetes in cancer patients [12]. However, the lack of association in our study may be attributed to underreporting of chemotherapy use [36]. Future studies with more comprehensive data on treatment regimens may help clarify the role of chemotherapy in type II diabetes risk among lung cancer survivors.

This study has some limitations. First, coding errors in the database, particularly in the ICD codes related to diabetes, may introduce non-differential misclassification. Since misclassification is unlikely to be differential by race and ethnicity groups, the overall findings could be biased towards the null. Obesity at cancer diagnosis was identified using ICD diagnosis codes in Medicare claims. We expect an underestimation of assessing obesity among both ANHPI and NHW lung cancer survivors. Noone et al. compared SEER treatment data with Medicare claims and reported moderate sensitivity of SEER data for identifying chemotherapy (68%) and radiation (80%). However, the sensitivity did not vary between ANHPI and NHW cancer patients. We expect that the percentages of lung cancer survivors receiving chemotherapy and radiation are underreported [36]. Second, the exclusion of patients enrolled in HMO due to the unavailability of claims data for this version of the data may potentially impact the generalizability of the findings; however, the remaining sample had a large sample size and high data quality. Lastly, the risk of type II diabetes among ANHPI lung cancer survivors may be underestimated if ANHPI lung cancer survivors were less likely to seek care or be diagnosed for diabetes compared to NHW lung cancer survivors.

Based on our knowledge, this study is the first study that examined the risk of type II diabetes after cancer diagnosis among ANHPI lung cancer survivors in comparison with NHW lung cancer survivors. One of the strengths of our study is that this is a large cohort study with 3920 older ANHPI lung cancer survivors with more than 20 years of follow-up. Furthermore, we disaggregated the ANHPI subgroup as allowed by the sample size. We obtained information on the incident diabetes diagnosis from the SEER-Medicare database, reducing recall bias and survivor bias in our study.

In conclusion, we observed a higher risk of incident type II diabetes among older Asian and NHPI lung cancer survivors compared to older NHW lung cancer survivors. Also, the prevalence of diabetes in ANHPI lung cancer survivors was higher in comparison with NHW lung cancer survivors. Additionally, obesity was a risk factor for type II diabetes among ANHPI lung cancer survivors. These findings suggest increasing surveillance on type II diabetes and monitoring body weight for ANHPI lung cancer patients may be beneficial, especially among high-risk subgroups such as Asian Indian, Pakistani, and Vietnamese patients. Additionally, personalized lifestyle interventions, including dietary counseling, physical activity promotion, and weight management programs, may help to mitigate diabetes risk and improve long-term health outcomes. Further studies are needed to explore other risk factors of type II diabetes among ANHPI lung cancer survivors.

## Supplementary Information

Below is the link to the electronic supplementary material.

Supplementary file1 (XLSX 28 KB)

Supplementary file2 (DOCX 120 KB)

## Data Availability

The datasets used to conduct this study are available upon approval of a research protocol from the National Cancer Institute. Instructions for obtaining these data are available at https://healthcaredelivery.cancer.gov/seermedicare/obtain/.
