# Supplementary material for: Increased risks of type II diabetes among older Asian, Native Hawaiian, and Pacific Islander lung cancer survivors: a SEER-Medicare analysis
Source: J Cancer Surviv. 2025 Mar 29;20(Suppl 1):40–9. doi: 10.1007/s11764-025-01786-5 (PMC13676505; doi:10.1007/s11764-025-01786-5)
Supplement: Supplementary file 1 [file 11764_2025_1786_MOESM2_ESM.docx]

# **Supplemental Figures**

Asian, NHPI, and NHW lung cancer survivors (N=323,543)

N=76,882

- Excluded patients who were identified by autopsy or death certificate (N=373)
- Excluded patients with unusual histology type at cancer diagnosis (N=601)
- Excluded patients who were not continuously enrolled in Medicare Part A or Part B or enrolled in HMO from one year prior to cancer diagnosis to the end of SEER follow-up period (N=120,883)
- Excluded patients with follow-up year <= 1 year (N=124,804)

ANHPI lung cancer survivors (N=3,920)

NHW lung cancer survivors (N=11,760)

Matched 3 NHW patients to 1 ANHPI patient by sex, cancer diagnosis year (±1), and cancer diagnosis age (±1)

ANHPI lung cancer survivors

(N=<3,930^a^ )

NHW lung cancer survivors

(N=<72,960^a^ )

- Excluded ANHPI patients who did not have matched NHW patients or patients who only had one matched NHW patients (N=<11^a^)
- Excluded NHW patients who did not have at least one matched ANHPI patient (N=<61,200^a^ )

## **Supplemental Figure 1. Study inclusion/exclusion criteria.**

## Abbreviations: NHPI, Native Hawaiian and Pacific Islander; ANHPI, Asian, Native Hawaiian and Pacific Islander; NHW, Non-Hispanic White; HMO, Health Maintenance Organization. a. counts (<11) and % were suppressed per U.S. Department of Health and Human Services CMS cell size suppression policy. Additional cells are suppressed so suppressed numbers cannot be calculated.

**Supplemental Figure 2. Cancer treatment of lung cancer survivors diagnosed from 2000-2017 in the SEER Medicare data, stratified by race and ethnicity.**
